# Reducing Delays in Diagnosing Primary Immunodeficiency Through the Development and Implementation of a Clinical Decision Support Tool: Protocol for a Quality Improvement Project

**DOI:** 10.2196/32635

**Published:** 2022-01-04

**Authors:** Bharat Kumar, Samuel Zetumer, Melissa Swee, Ellen L Keyser Endelman, Manish Suneja, Benjamin Davis

**Affiliations:** 1 Division of Immunology Department of Internal Medicine University of Iowa Carver College of Medicine Iowa City, IA United States; 2 Department of Internal Medicine University of Iowa Carver College of Medicine Iowa City, IA United States; 3 Division of Nephrology Department of Internal Medicine University of Iowa Carver College of Medicine Iowa City, IA United States; 4 Chicago Theological Seminary University of Chicago Chicago, IL United States

**Keywords:** immunology, clinical decision support, diagnostic decision-making

## Abstract

**Background:**

Primary immunodeficiencies (PIs) are a set of heterogeneous chronic disorders characterized by immune dysfunction. They are diagnostically challenging because of their clinical heterogeneity, knowledge gaps among primary care physicians, and continuing shortages of clinically trained immunologists. As a result, patients with undiagnosed PIs are at increased risk for recurrent infections, cancers, and autoimmune diseases.

**Objective:**

The aim of this research is to develop and implement a clinical decision support (CDS) tool for the identification of underlying PIs.

**Methods:**

We will develop and implement a CDS tool for the identification of underlying PIs among patients who receive primary care through a health care provider at the University of Iowa Hospitals and Clinics. The CDS tool will function through an algorithm that is based on the Immune Deficiency Foundation’s 10 Warning Signs for Primary Immunodeficiency. Over the course of a year, we will use Lean Six Sigma principles and the Define, Measure, Analyze, Improve, and Control (DMAIC) framework to guide the project. The primary measure is the number of newly diagnosed PI patients per month. Secondary measures include the following: (1) the number of new patients identified by the CDS as being at high risk for PI, (2) the number of new PI cases in which immunoglobulin replacement or rotating antibiotics are started, (3) the cost of evaluation of each patient identified by the CDS tool as being at high risk for PIs, (4) the number of new consults not diagnosed with a PI, and (5) patient satisfaction with the process of referral to the Immunology Clinic.

**Results:**

This study was determined to not be Human Subjects Research by the Institutional Review Board at the University of Iowa. Data collection will begin in August 2021.

**Conclusions:**

The development and implementation of a CDS tool is a promising approach to identifying patients with underlying PI. This protocol assesses whether such an approach will be able to achieve its objective of reducing diagnostic delays. The disciplined approach, using Lean Six Sigma and the DMAIC framework, will guide implementation to maximize opportunities for a successful intervention that meets the study’s goals and objectives as well as to allow for replication and adaptation of these methods at other sites.

**International Registered Report Identifier (IRRID):**

PRR1-10.2196/32635

## Introduction

Primary immunodeficiencies (PIs) are diagnostically challenging chronic disorders involving the immune system that lead to recurrent infections, increased risk for cancer, and autoimmune disease [1]. PIs may present at any age and manifest due to genetic predilection and environmental exposures [2].

Diagnoses of PIs are frequently delayed because of this clinical heterogeneity, knowledge gaps among primary care physicians, and continuing shortages of clinically trained immunologists [3-5]. These delays in obtaining care lead to increased mortality and morbidity as well as decreased quality of life [6-9].

PI diagnostic delays also cost a calculated $85,882 per patient [10] and contribute $40 billion in costs to the US health care system. It has been estimated that early diagnosis can save $6500 per patient [11]. Earlier identification of PI is critical for the following reasons:

1. To permit more appropriate management of the underlying condition. Immune globulin replacement and immunomodulators have demonstrated efficacy in reducing the frequency and severity of infections [12,13].
2. To enable more appropriate preventative health measures. Guidelines from the Advisory Committee on Immunization Practices (ACIP) and Infectious Disease Society of America (IDSA) state that immunocompromised patients, including those with PIs, have different requirements for vaccinations [14,15]. Identification of PIs enables more timely vaccinations and prevention of illness. Conversely, certain live vaccinations are contraindicated in certain types of PIs and should be avoided to prevent adverse events [16]. Finally, those with PI are at 1.4 to 1.6-fold increased risk for malignancy, with some estimates of prevalence of malignancy as high as 25% [17,18]. Therefore, a diagnosis of a PI significantly impacts screening guidelines for malignancy.
3. To prevent downstream diagnostic errors. Patients with a PI are more likely to present with atypical manifestations of infections that are less likely to be identified early on. Local infections are also more likely to become systemic, leading to increased morbidity and mortality [19]. Recognition of PIs enables providers to consider these diagnoses earlier and prevent further diagnostic delays. Studies also demonstrate that early diagnosis prevents further readmissions and improves Accountable Care Organization (ACO) scores [20].
4. To uncover diagnoses of family members. PIs are inherited conditions [21]. Identification of a proband in a family with immune deficiency enables family members who have compatible signs and symptoms to share information with one another.
5. To empower patients about chronic illness. A diagnosis of PI is empowering to patients and enables them to reach out to support systems. Studies have demonstrated that early detection and diagnosis improves patient satisfaction and quality of life [22].
6. To address health disparities. PIs are more likely to affect patients in particular ethnic populations, including those of Middle Eastern descent [23]. Experts also voice concern that diagnostic delays disproportionately affect Black, Indigenous, and other people of color (BIPOC) and those with already reduced access to health care [24,25]. Additionally, PIs affect women in specific ways that amplify the downstream effects of diagnostic delays, including gynecologic and obstetric complications [26].

## Methods

### Objectives and Specific Aims

The objective of this study is to develop and implement a clinical decision support (CDS) tool for identifying patients with an underlying PI. The Jeffrey Modell Foundation has published a set of 10 warning signs for PIs for both adults and children [27]. We will adapt these warning signs to a CDS tool to help stratify the risk of a patient having an underlying PI. This CDS tool will enable primary care providers within the University of Iowa to refer patients to the University of Iowa’s Immunology Clinic, and guide laboratory investigations prior to referral to ensure readiness prior to the visit.

Toward that end, there are two specific aims:

1. Identify the total number of patients who present to the University of Iowa General Internal Medicine, Pediatrics, or Family Medicine clinics with two or more warning signs of a PI over a 12-month period.
2. Refer at least 10% of these identified patients to a University of Iowa clinical immunologist for evaluation within 12 months of their initial presentation.

### Rationale for the Intervention

CDS tools for PIs have not yet been developed and assessed in peer-reviewed literature. This intervention would represent the first attempt to address this critical need. However, we build upon several lines of evidence that bolster the rationale that a CDS tool is needed to identify PI patients:

1. A 2019 study of a large pediatric cohort of 185,892 patients revealed 2188 patients (1.26%) at medium to high risk for PIs [28]. The authors concluded that early identification of PIs in the 98 patients who were at highest risk within this cohort would represent an annual cost savings of up to $7.7 million.
2. CDS tools for identifying secondary immunodeficiencies, including HIV, have been developed. Although these methods are different and guidelines for implementation vary considerably, they add to the evidence of the feasibility of immunodeficiency screening of large populations [29].
3. Several CDS tools have been developed for tracking antibiotic usage, including at the University of Iowa [30]. The purposes of these CDS tools vary, but collection of data about the frequency of use and type of antibiotic is vital in this proposal, further supporting the feasibility of our approach.

### Settings and Target Population

University of Iowa Hospitals and Clinics (UIHC) represents the only comprehensive academic medical center within the state of Iowa. Between July 1, 2019, and June 30, 2020, there were 1,039,681 clinic visits, 32,872 inpatient admissions, and 50,468 emergency department visits. In the UIHC system, there are 77,779 patients that have a primary care provider in Family Medicine, General Internal Medicine, and General Pediatrics.

Patients with suspected immunodeficiencies are evaluated at the Allergy and Immunology Clinic located at the University of Iowa’s clinics in Iowa City. If patients are determined to have an immunodeficiency, then they return to the Allergy and Immunology Clinic to obtain specialized care. Per year, there are 1863 half-day clinics staffed by 12 physicians serving 8963 patients. There are approximately 1684 patients with a diagnosis of a PI whose management is overseen by physicians and other practitioners in the Immunology Clinic. The average wait time to a new appointment is 5.46 weeks for adults and 13.16 weeks for pediatric patients.

### Inclusion and Exclusion Criteria

Inclusion criteria include patients who receive their care from one of the General Internal Medicine, Pediatrics, or Family Medicine clinics through the UIHC system. Exclusion criteria include patients with an established secondary or acquired immunodeficiency, and patients with an established PI diagnosis.

### Quality Improvement Framework

We will use Lean Six Sigma principles and the Define, Measure, Analyze, Improve, and Control (DMAIC) framework to implement this intervention. The Lean Six Sigma approach has been previously described in health services research literature as a methodology to improve health care delivery [31]. It combines two distinct process improvement methodologies: Lean and Six Sigma. The Toyota Motor Corporation initially developed Lean to improve manufacturing quality through the elimination of waste and creation of value for customers [32]. Lean complements Six Sigma, a data-driven management approach aimed at the elimination of defects and reduction of unwarranted variability [33].

### Define Phase

**Figure 1:**
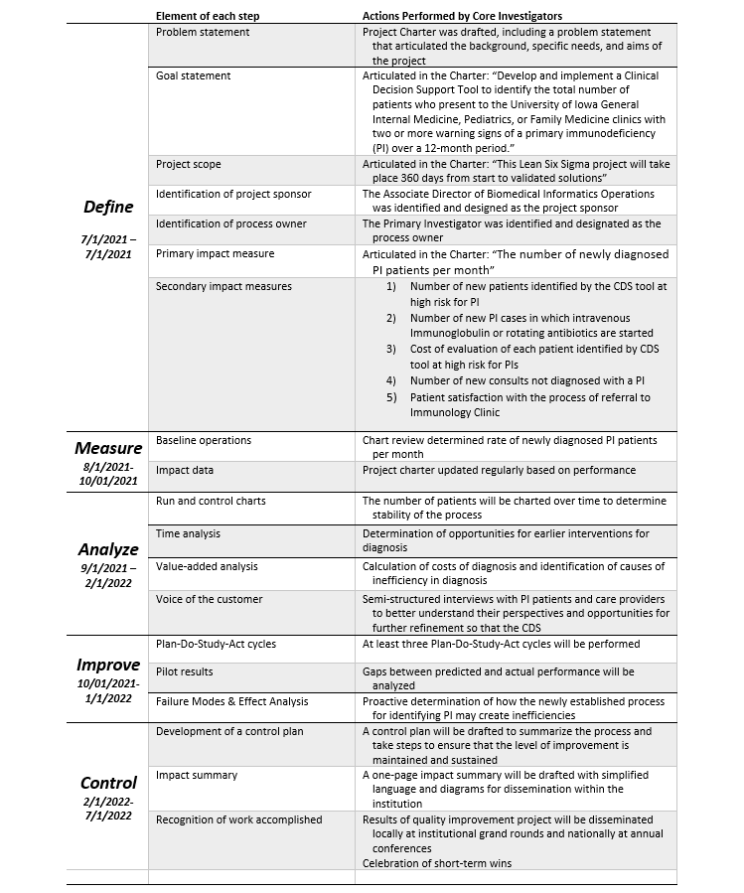
The Define, Measure, Analyze, Improve, and Control (DMAIC) framework. CDS: clinical decision support; PI: primary immunodeficiency.

**Figure 2:**
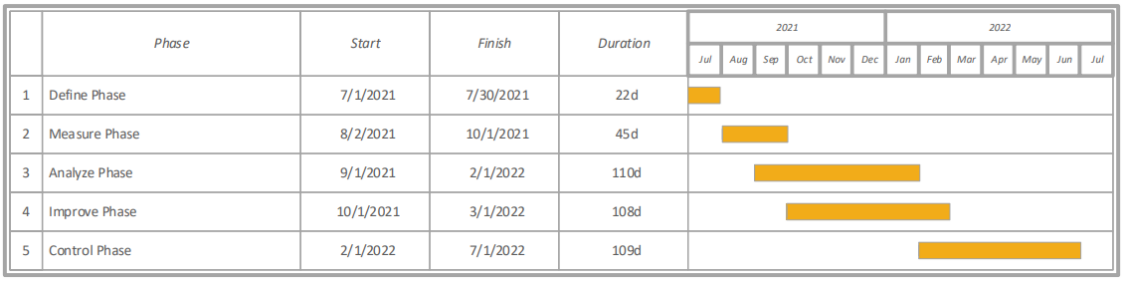
Gantt chart outlining major phases of the study protocol.

In line with the DMAIC framework (Figure 1), we have begun defining the scope of the project. We have developed a project charter to articulate the problem at hand, our project goals, and customer requirements. The project charter also delineates the time frame for completion of tasks (Figure 2).

Additionally, to understand the needs of the customers, we are using Voice of the Customer to clarify experiences, expectations, and needs. We are currently conducting semistructured interviews with 20 patients with PIs to understand their experiences. EK, a core member of the team, is a patient with PI and is helping to formulate questions and analyze data.

### Measure Phase

During the Measure phase, we anticipate collection of data regarding our primary and secondary outcomes. The primary measure, as articulated in the project charter, is the number of newly diagnosed PI patients per month. We will measure the rate over the past 24 months to obtain a baseline, and then monitor it throughout the course of the protocol.

We will also measure the following five secondary measures:

1. Number of new patients identified by the CDS tool as being at high risk for PI.
2. Number of new PI cases in which immunoglobulin replacement or rotating antibiotics are started.
3. Cost of evaluation of each patient identified by CDS tool as being at high risk for PIs.
4. Number of new consults not diagnosed with a PI.
5. Patient satisfaction with the process of referral to the Immunology Clinic.

Administrative data will be used for the primary measure and secondary measures 1-4. For secondary measure 5, we will conduct semistructured interviews and surveys to obtain the Voice of the Customer.

### Analyze Phase

Throughout the analyze phase, we will use the data being obtained as part of the Measure phase to assess the efficacy of the intervention and to guide modifications as necessary.

For quantitative data, we will construct both run and control charts. The specific type of chart will be dependent on the number of evaluated patients and specific outcome measures. For example, if the CDS tool is unable to find many patients with PI, then a T-chart may be more appropriate than an X-bar chart.

For qualitative data, we will continue to use Voice of the Customer. Once the CDS tool is implemented, we will conduct semistructured interviews with primary care providers to better understand their perspectives and opportunities for further refinement so that the CDS tool is more user-friendly.

For individual cases of newly diagnosed PI, we will perform more detailed analysis to recognize causes of diagnostic delays. We will use value-added analysis to calculate the costs of diagnosis and identify causes of inefficiency in diagnosis. Additionally, we will use time analysis to chronologically map out opportunities for earlier diagnosis. Both of these analyses will then be used to further refine the CDS tool.

### Improve Phase

During the Improve phase, we will employ sequential Plan-Do-Study-Act (PDSA) cycles to refine the CDS tool to different settings, starting with Internal Medicine, then Pediatrics, and finally Family Medicine. The investigators will continue to use Voice of the Customer as a key Lean Six Sigma tool to guide these PDSA cycles.

Additionally, as we begin to finalize the CDS tool, we will use failure mode and effect analysis to proactively determine how the newly established process for identifying PIs may create inefficiencies and result in failures in diagnosis.

### Control Phase

Toward the end of the quality improvement project, we will develop a monitoring and proactive response plan to sustain the improvement plan. Using those measures, we will establish a trigger level to ensure that new cases of suspected PIs are screened through the CDS tool. Additionally, with advances in the diagnosis of PIs, such as genetic testing, we will incorporate newer modes of evaluation into the CDS tool.

Finally, we intend on publishing our findings and presenting them at national meetings. Thorough documentation of the process, including this protocol, will be essential for transfer of knowledge to other settings and implementation in different settings.

### Ethics Approval and Consent to Participate

This study protocol was submitted to the University of Iowa Institutional Review Board and determined not to be Human Subjects Research.

## Results

As of July 31, 2021, this protocol has been funded by the Society to Improve Diagnosis in Medicine as a DxQI Grant. It has been determined to not be Human Subjects Research by the University of Iowa Institutional Research Board on July 13, 2021. Data collection will begin on August 15, 2021, and end on July 31, 2022. We intend on publishing results in winter 2022.

## Discussion

While there have been tremendous advances in the modalities used to diagnose PIs, evidence of real-world interventions to better identify PI are largely lacking. To fill that evidence gap, this study protocol outlines a quality improvement initiative to help reduce delays in diagnosing PIs. To bolster the likelihood of success, we have designed our initiative in line with the principles of Lean Six Sigma and have structured it via the DMAIC framework.

We anticipate a series of challenges and have a comprehensive strategy to address these challenges as they arise. First, we have a screening algorithm (Multimedia Appendix 1) that has multiple redundancies for optionality if one method of obtaining data cannot be completed properly.

Second, we have a plan to accommodate increased volumes of patients who would need evaluation. Currently, there are 1863 half-day immunology clinics among 12 faculty members serving 8963 unique patients. The average wait time to a new appointment is 5.46 weeks for adults and 13.16 weeks for pediatric patients. The division has plans to expand with the hiring of 1-2 new faculty members over the coming academic year. Additionally, as allergy/immunology faculty are certified to see both adult and pediatric patients, flexibility can be arranged to accommodate incoming evaluations.

Third, the CDS tool will be implemented in a stepwise pattern through the clinics to assess its ability to achieve our stated specific aims. Therefore, we have the capacity to revise the tool accordingly as we recognize the specificity and sensitivity of elements within the algorithm in detecting cases of PI.

## Appendix

Multimedia Appendix 1 Primary immunodeficiency screening algorithm.

Multimedia Appendix 2 Peer review reports from the Society to Improve Diagnosis in Medicine.

## Abbreviations

ACIP: Advisory Committee on Immunization Practices
ACO: Accountable Care Organization
BIPOC: Black, Indigenous, and other people of color
CDS: clinical decision support
DMAIC: Define, Measure, Analyze, Improve, and Control
IDSA: Infectious Disease Society of America
PDSA: Plan-Do-Study-Act
PI: primary immunodeficiency
UIHC: University of Iowa Hospitals and Clinics
