# Supplementary material for: Reducing Delays in Diagnosing Primary Immunodeficiency Through the Development and Implementation of a Clinical Decision Support Tool: Protocol for a Quality Improvement Project
Source: JMIR Res Protoc. 2022 Jan 4;11(1):e32635. doi: 10.2196/32635 (PMC8767470; doi:10.2196/32635)
Supplement: Multimedia Appendix 1 [file resprot_v11i1e32635_app1.docx]

**Two-Part Immunodeficiency Clinical Decision Support Algorithm (ICDSA)**

Once the inclusion/exclusion criteria are applied and the record is pre-processed, the ICDSA will be used to determine whether a patient has two or more warning signs and would require evaluation by a clinical immunologist. Based on the Jeffrey Modell Foundation’s 10 Warning Signs for Primary Immunodeficiency, each warning sign has its own sub-algorithm that determines whether the patient has met criteria for that warning sign.


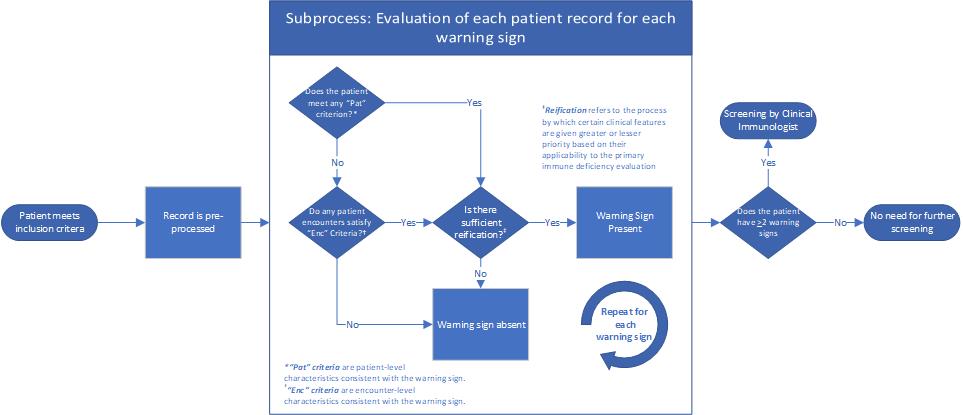


| Warning Sign | Part 1: Patient-Level or Encounter-Level Case Identification | Part 2: Encounter or Patient Reification |
| --- | --- | --- |
| 1. *Two or more new ear infections within one year.* | **Pat A:** Patients with a diagnosis of recurrent ear infection in their problem list.  **Pat B:** Patients with documented past surgical history or CPT codes consistent with surgical treatment for chronic/recurrent ear infections.  ***Enc A:*** *Encounters with ICD codes for ear infection listed in discharge summary problem list or billing codes for clinic visit.*  ***Enc B:*** *Encounters with ICD codes for ear infection affiliated with orders.*  ***Enc C:*** *Encounters with Otolaryngology Consult/Clinic in which the Otolaryngology note has multiple instances of the phrase “ear infection” or “otitis media”.* | **Increase Case Confidence:**  - Encounters during which the patient receives a full course of antibiotics for acute otitis media. (+2)  - Encounters not immediately preceding encounters identified as cases of ear infections. (+1)  - Encounters in which the phrase “acute otitis media” or “bacterial otitis media” occur repeatedly. (+2)  **Decrease Case Confidence:**  - Encounters in which no antibiotics were administered. (-2)  - Otitis media related ICD-10 codes are not affiliated with the encounter. (-3) |
| 2. *Two or more new sinus infections within one year, in the absence of allergy*. | **Pat A:** Patients with a diagnosis of recurrent sinus infection in their problem list.  **Pat B:** Patients with documented past surgical history or CPT codes consistent with treatment for chronic/recurrent sinus infections.  ***Enc A:*** *Encounters with ICD codes for sinus infections listed in discharge summary problem list or billing codes for clinic visit.*  ***Enc B:*** *Encounters with an Otolaryngology note containing the phrase “Sinus Infection” in the assessment.* | **Increase Case Confidence:**  - Encounters during which a full course of antibiotics were administered. (+2)  - Encounters with the phrase “sinus” and “sinus pain” documented in multiple notes (not just first note). (+2)  - Encounters with discharge summaries that contain the phrase “acute otitis media” or “bacterial otitis media. (+3)  - Encounters with positive sputum/nasal discharge cultures. (+1)  - Encounters with fever. (+1)  **Decrease Case Confidence:**  - Encounters during which no antibiotics were given. (-1) |
| 3. *One or more pneumonia per year for more than one year.* | **Pat A:** Patients with a diagnosis of recurrent pneumonia in problem list.  **Pat B:** Patients with > 2 outpatient Pulmonology clinic visits with phrase “pneumonia” listed in problem list visit.  ***Enc A:*** *Encounters with ICD codes for pneumonia listed in discharge summary problem list or billing codes for clinic visit.*  ***Enc B:*** *Encounters with ICD codes for pneumonia affiliated with orders written after the first day of admission.*  ***Enc C:*** *Encounters with a positive sputum culture (rule out contaminant with free text and/or speciation) and any of the following: elevated WBC, O2 requirement, or fever.* | **Increase Case Confidence:**  - Encounters during which the patient receives a full course of antibiotics for pneumonia. (+2)  - Encounters during which chest imaging report contains the phrase “consolidation” or “infection” in it. (+2)  **Decrease Case Confidence:**  - Encounters during which the patient receives a full course of steroids. (-2)  - Encounters with “COPD exacerbation” as a diagnosis listed in the problem list in discharge summary, or affiliated with orders written after the first day. (-2)  - Patients with a diagnosis of COPD in their problem list. (-1)  - Encounters following encounters during with COPD exacerbation as a primary diagnosis in the discharge summary. (-1) |
| 4. *Chronic diarrhea with weight loss.* | **Pat A:** Prior to encounter identification or further patient identification, the patient’s EMR is screened for the following: Any three consecutive weights > 10kg lower than any prior weight, or ICD codes for weight loss listed in their problem list. If the patient does not meet those criteria, no further case identification is necessary. If the patient meets one or more of those criteria, the following criteria are applied to patients and/or encounters for further case identification:  **Pat A1:** Patients with a diagnosis of “chronic diarrhea” or “recurrent diarrhea” listed as a problem in their problem list.  **Pat A2:** Patients with multiple gastroenterology clinic visits with the phrase “diarrhea” in the clinic note.  ***Enc A:*** *Encounters with ICD codes for diarrhea/chronic diarrhea/C-diff/gastroenteritis listed in the discharge summary problem list or as billing codes for orders during clinic visits.*  ***Enc B:*** *Encounters with the phrase “diarrhea” in H&P and > 1 progress note.*  ***Enc C:*** *Encounters with either positive stool cultures, or stool labs with the phrase “diarrhea” or “gastroenteritis” in any notes affiliated with that encounter.* | **Increase Case Confidence:**  - Patients with a future weight > 20kg lower than any past weight. (+1)  - Encounters with a positive stool culture, not listed as a contaminant. (+2)  **Decrease Case Confidence:**  - Encounters with diagnoses associated with chronic diarrhea (eg. pancreatic insufficiency) that are not also associated with immunodeficiency as primary diagnoses. *Note: some immunodeficiencies are associated with diarrhea, but this association is NOT mediated by an increased likelihood of infectious diarrhea. For example, some autoimmune conditions are associated with biliary disease, which can itself be associated with diarrhea but not gastroenteritis.* (-2)  - Patients with a diagnosis of IBD (Crohn’s or Ulcerative Colitis) or IBS in their problem list. (-1) |
| 5. *Recurrent viral infections (colds, herpes, warts, condyloma).* | **Pat A:** Patient’s problem list contains > 1 ICD code matching a viral infection: viral gastroenteritis, condyloma, rhinosinusitis etc.  **Pat B:** Patient’s problem list contains “recurrent X”, where X is one of the problems associated with viral infections.  ***Enc A:*** *Encounters with primary problems matching any of the ICD-10 codes matching a viral infection: viral gastroenteritis, condyloma, rhinosinusitis etc.*  ***Enc B:*** *Encounters with Dermatology (either inpatient consult or clinic) for which any diagnostic code or associated ICD-10 codes match any of the above viral infections that have skin findings.*  ***Enc C:*** *Encounters with Otolaryngology for which any diagnostic code or associated ICD-10 codes match the above viral infections that manifest in the ears, nose, or throat.* | **Increase Case Confidence:**  - Patients with encounters that meet > 1 encounter type, eg, an encounter meeting Enc A criterion and a separate encounter meeting Enc B criterion. (+2) |
| 6. *Recurrent need for intravenous antibiotics to clear infections.* | **Pat A:** Patients with > 2 outpatient Infectious Disease clinic visits during which the patient was on an intravenous antibiotic.  ***Enc A:*** *Encounters in which Infectious Disease was consulted and whose last note contains the phrase “OPAT” (OutPatient Antibiotic Therapy).*  ***Enc B:*** *Encounters during which or after which antibiotics are administered for more than two weeks unless the encounter during which the antibiotics were started had diagnostic codes for infective endocarditis or other infections for which the recommended antibiotic duration is two or more weeks.*  ***Enc C:*** *Patients whose antibiotic duration is 1.25 times as long as the maximum recommended antibiotic duration to treat the infection associated with the encounter during which the order for the antibiotic was written.* | **Increase Case Confidence:**  - Score the total number of days for which the patient was given a prescription for antibiotics, and divide by 25. (+[IV antibiotic duration] / 25)  - Patients whose IV antibiotic duration is > 50% of their total antibiotic duration.  **Decrease Case Confidence:**  - Patients for whom the antibiotic duration for each intravenous antibiotic administration was equal to or less than the recommended antibiotic duration of the infection whose diagnostic code is associated with the encounter during which the antibiotic was first administered. (-1)  - Patients with a diagnosis of infective endocarditis. (-1) |
| 7. *Recurrent, deep abscesses of the skin or internal organs.* | **Pat A:** Patients with ICD-10 codes associated with recurrent abscess in their problem list.  **Pat B:** Patients with multiple CPT codes for incision and drainage affiliated with their past surgical history.  ***Enc A:*** *Encounters with associated CPT codes for incision and drainage.*  ***Enc B:*** *Encounters in which cultures were collected from an identified sample source labelled “abscess”.*  ***Enc C:*** *Encounters with abscess as any problem associated with the discharge summary.*  ***Enc D:*** *Encounters with imaging reports containing the phrase “fluid collection” or “abscess”. Reserve this criteria for patients who already have other encounters that have met other criteria.* | **Increase Case Confidence:**  - For encounters with an incision and drainage operation associated with them, inclusion of the word “purulent” in the operative report. (+2)  - Encounters for which the phrase “abscess” is contained within the primary problem. (+3)  - If location data is available in the problem list or after parsing the radiological report, abscesses in a different location than prior abscesses result in an increase in case confidence. (+1)  **Decrease Case Confidence:**  - Available cultures from a source labelled “abscess” are negative, ie do not grow anything and have nothing on stain. (-1)  - Encounters in which no surgery, interventional radiology, or ultrasound consults were placed. (-1) |
| 8. *Persistent thrush or fungal infections of the skin or internal organs*. | **Pat A:** Patients with two or more different medical problems associated with fungal illness, eg. a patient with oral thrush *and* Blastomyces dermatitis.  **Pat B:** Patients with a diagnosis of “recurrent fungal infection” in their problem list.  ***Enc A:*** *Encounters in which there is a positive fungal culture without reference to a contaminant, or another fungal assay returns positive.*  ***Enc B:*** *Encounters in which antifungals were given.*  ***Enc C:*** *Encounters for which a fungal infection was among one of the problems in the discharge summary.* | **Increase Case Confidence:**  - Encounters during which prescriptions of antifungals were written for longer durations than recommended for the fungal infections diagnosed during the encounter. (+1)  - Encounters during which both an antifungal prescription ends and another antifungal prescription is started. (+1)  - Patients with two different non-contaminant fungi growing among all the fungal cultures collected over all encounters. (+2)  - Encounters during which amphotericin B was prescribed. (+1) |
| 9. *Infection with normally harmless tuberculosis-like bacteria.* | **Pat A:** Patients with a diagnosis of “non-tuberculosis mycobacterium” as a medical problem.  **Pat B:** Patients with non-TB mycobacterium grown in any culture, or non-TB mycobacterium identified in acid-fast stain, or identified with DNA probe. | **Increase Case Confidence:**  - Patients for whom non-TB mycobacterium has been a primary diagnosis upon discharge. (+2)  - Patients for whom an ICD code for non-TB mycobacterium is associated with a prescription for an antimicrobial prescription. (+2)  **Decrease Case Confidence:**  - Patients whose cultures that grew mycobacterium have text containing the word “contaminant” in them. |
| 10. *A family history of PI* | **Pat A:** Given that this sub-algorithm is computationally expensive, a necessary criterion to further screen the patient for a family history of immunodeficiency is that the patient must have at least one other warning sign among warning signs 1) through 9). If the patient has no other warning signs, neither case identification nor reification should be performed.  **Pat A1:** Patients whose family history data contains the phrase “immunodeficiency.”  **Pat A2:** For each *other* patient who either has a diagnosis of primary immunodeficiency *or whom this algorithm identifies as having* > *2 warning signs,* identify their relatives as having 1 warning sign.  **Pat A3:** For every encounter meeting one or more Enc criteria, scan all notes for phrases similar to “family history of immunodeficiency” | **Increase Case Confidence:**  - Positive reification for patients whose family members have > 2 warning signs (Pat B criterion) increases with an increasing number of warning signs and degree of positive reification in that family member/relative. (+ 1 to 3).  **Decrease Case Confidence:**  - Family members who both do not have a diagnosis of primary immunodeficiency and who have no warning signs per their own EMR. (-1) |
