# Supplementary material for: Reducing Delays in Diagnosing Primary Immunodeficiency Through the Development and Implementation of a Clinical Decision Support Tool: Protocol for a Quality Improvement Project
Source: JMIR Res Protoc. 2022 Jan 4;11(1):e32635. doi: 10.2196/32635 (PMC8767470; doi:10.2196/32635)
Supplement: Multimedia Appendix 2 [file resprot_v11i1e32635_app2.pdf]

DxQI SGP Grant Review Summary Report

Grant No: 382

Project Title: Reducing Delays in Diagnosing Primary Immunodeficiency Through the Development and Implementation of a Clinical Decision Support Tool

Total Points after Peer Review (282 possible): _269_

Peer Reviewer Comments:

Reviewer 1:
No Comments

Reviewer 2:
No Comments

Reviewer 3:
No Comments

Patient & Family Engagement Reviewer Comments:

Reviewer 1:
Voice of Customer used to clarify the experience of patients with PI in a collaborative effort between patients and providers to bring these voices to the forefront - enables identification of opportunities for early ID of PIs
Also measuring patient satisfaction with referral to the immunology clinic through surveys and interviews
Use Voice of Customer to refine the tool with each iteration of the cycle
Core team member is also a PI patient, lending patient voice to the project
Project idea is an outgrowth of a patient-partnered needs assessment

Comments from the Grant Review Committee:

Reviewer 1:
Aims, background rationale and approach described in sufficient detail. Team as described has the expertise required to meet the project goals. Additional insight into how the patient representative member of team will elicit real-time feedback from patients would be of interest. Good case is made for this project advancing evidence-based practice in diagnostic safety, including literature cited. The tool should be replicable/reproducible, and the process applied to other diagnostic improvement needs. Methodology and time frame (and discussion of how anticipated challenges will be managed) support accomplishment of goals as outlined.

Reviewer 2:

DxQI SGP Grant Review Summary Report

The concept of using CDS to decrease delay in diagnosis and thus potentially improve time to treatment, appropriate treatment and referral and mitigation of downstream costs is important. This project would build from a previous effort to develop the algorithm for building the Primary Immunodeficiency CDS tool. The investigators have a well designed approach for consumer (patient and family engagement). This project should advance innovative opportunities in evidence-based practice that would impact the diagnostic journey at least for patients with PI. It's unclear if the knowledge gained would translate to other conditions, but plausible to think in this direction. No discussion of scalability beyond University of Iowa.

Feedback from the Grant Review Committee meeting:

- Thank you for your outstanding proposal.
- Consider keeping track of costs of screening to add to the understanding of financial feasibility in a population. Including non-monetary costs of screening such as inducing anxiety, would also be useful.
- Consider how this might be scaled beyond a single health system if it is successful.
